# A 3D star shot to determine the gantry, collimator, and couch axes positions

**DOI:** 10.1002/acm2.13623

**Published:** 2022-04-29

**Authors:** Robert Corns, Kaida Yang, Mason Ross, Shiva Bhandari, Makunda Aryal, Peter Ciaccio

**Affiliations:** ^1^ Department of Radiation Oncology Brody School of Medicine East Carolina University Greenville North Carolina USA; ^2^ Department of Physics East Carolina University Greenville North Carolina USA

**Keywords:** 3D star shot

## Abstract

A linear accelerator has three independent axes that are nominally intersecting at the isocenter. Modern treatment techniques require the coincidence of these axes to lie within a 1‐mm diameter sphere. A solution to verify this requirement is to wrap a film on a cylindrical surface, align the cylinder to the linac's isocenter and gantry axis, and take multiple exposures of slits, rotating either the gantry, collimator, or couch between exposures. The resulting exposure pattern is the 3D equivalent of the 2D star shot and encodes sufficient information to determine each axis’ position in 3D. Moreover, this method uses a single sheet 8“x10” film, a standard film scanner, and a phantom that can be readily built in‐house, making a practical solution to this 3D‐measurement problem.

## 1 INTRODUCTION

Determination of the isocenter size and position is part of any quality assurance (QA) program for external beam radiation therapy.^1^ Modern treatment techniques require coincidence of these axes lie within a 1‐mm diameter sphere. A stable isocenter is important for stereotactic radiosurgery (SRS) and stereotactic body radiation therapy (SBRT). These techniques use tightly conformal beams directed toward a small target volume whose diameter is 1–3 cm. High spatial accuracy is critical to these treatments because they deliver large doses in a single or hypofractioned sessions.^2^ Isocenter displacements of 1–3 mm have significant impact because the tumor volumes are relatively small. Linac rotations have small mechanical shifts but when these shifts cause less than a 1 mm uncertainty in the target position, the changes in dosimetry are not clinically significant. However, discrepancies larger than 1 mm are not acceptable as they may lead to severe side effects.^3^ Wang has reported that 2‐mm positioning errors in spine SRS treatments lead to 5% or greater losses in tumor coverage, and more than 25% dose increases to healthy tissues.^4^ Guckenberger has also reported that each spatial error of 1 mm can decrease target coverage by 6% and dose conformity by 10%.^5^

The current American Association of Physicists in Medicine (AAPM) recommendation^6^ for the coincidence of radiation and mechanical isocenter is ±1 mm from baseline for an SRS/SBRT‐designated linac and ±2 mm from baseline for an Intensity Modulated Radiotherapy (IMRT) linac. More recently, the Canadian Partnership for Quality Radiotheraphy (CPQR) has recommended confirming the couch, collimator, and gantry axes lie near each other within a sphere of diameter 1 mm.^7^ It is anticipated that locating the isocenter in three dimensions will become a widely accepted requirement for robust QA programs.

Several techniques have been used to locate the isocenter. A traditional star shot is a two‐dimensional method, taking multiple exposures on either a film or a portal imager. Each exposure is of a radiation slit, and the slit is rotated between shots. The axis of rotation is characterized by the smallest circle that touches the midline of each slit exposure.^8^ The 2D star shot lacks the ability to relate all three axes to each other. Another widely used method of isocenter confirmation is the Winston‐Lutz test.^9^ This involves imaging a ball bearing inside a small radiation field with images taken over multiple gantry, couch, and collimator angles. Multiple methods have been proposed to measure the isocenter location in three dimensions, namely PRESAGE,^10^ NIPAM kV‐CBCT,^11^ and a film stack phantom.^12^ This paper introduces a method of radiation and mechanical isocenter verification using a single film. This is accomplished by wrapping a film around a cylinder and then aligning the cylinder axes to the mechanical axes of the linear accelerator. Next, slit exposures are taken over varying gantry, collimator, and couch angles. The images are analogous to 2D star shot films except wrapping film on a cylinder allows one to extend this concept to 3D. Hence, it shall be referred to as a 3D star shot. An advantage of this method is it utilizes a single sheet of film per study that can be scanned with a standard film scanner and analyzed.

## 2 METHODS

### 2.1 Concepts

**FIGURE 1:**
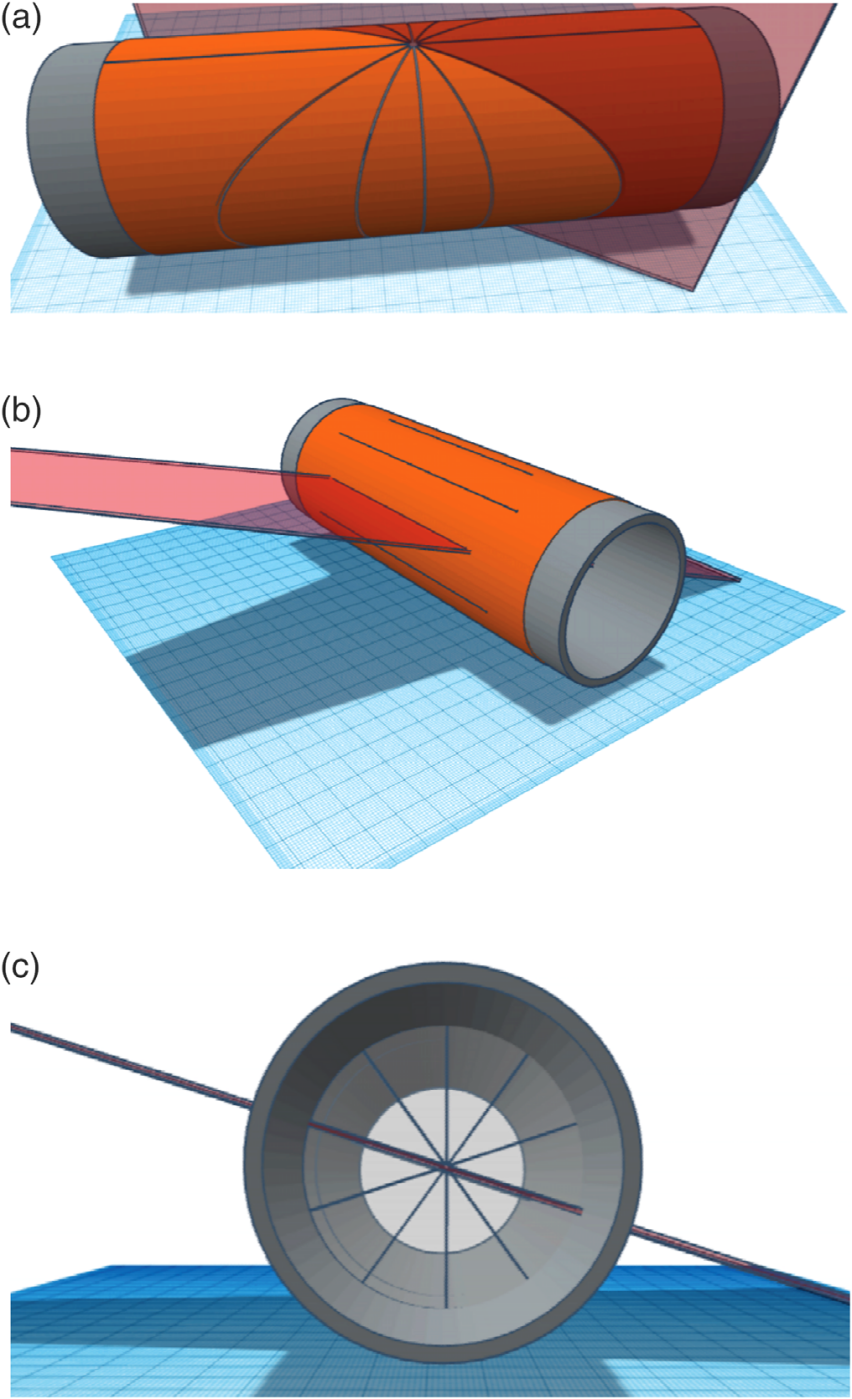
(a) The couch and collimator exposures form the spokes of a star pattern at the entrance and exit of the radiation slit on the cylindrical film. The resulting curves on the film are cylindrical sections. (b) The gantry exposure form sets of lines on the film parallel to the cylindrical axis. The entrance lines will be shorter than the exit lines, and if the gantry angles are appropriately chosen, the entrance and exit lines alternate and are evenly spaced. (c) Looking end‐on, the spokes for the gantry exposures form a virtual star pattern. The darkened film marks the entrance and exit of each radiation slit. These patterns form all along the cylinder's axis

The method involves wrapping a film around a cylinder, aligning the cylinder axes up with the linac axes and taking multiple slit exposures. An axis is rotated separately between exposures while holding the other two axes at fixed values. A slit exposure represents a plane that cuts through the cylinder, and the image on the exposed film forms a curve described by a cylindrical section. Analyzing these exposures gives the necessary information to represent all three axes of rotation in 3D. These exposures are illustrated in Figure 1. The couch and collimator form a star shot pattern at the entrance and exit exposures. The characteristic star shot pattern appears because the film is flat in a neighborhood of the axis of rotation. The gantry exposures form lines parallel to the axis of the cylinder. The lines on the film will alternate as short and long segments, corresponding to the entrance and exit exposures, respectively. A star shot forms virtually on any axial slice of the cylinder. As viewed on an axial slice, the radiation slit marks an entrance and exit point, and these are sufficient to define a radial spoke. Collectively over all line pairs, a virtual star shot is formed.

**FIGURE 2:**
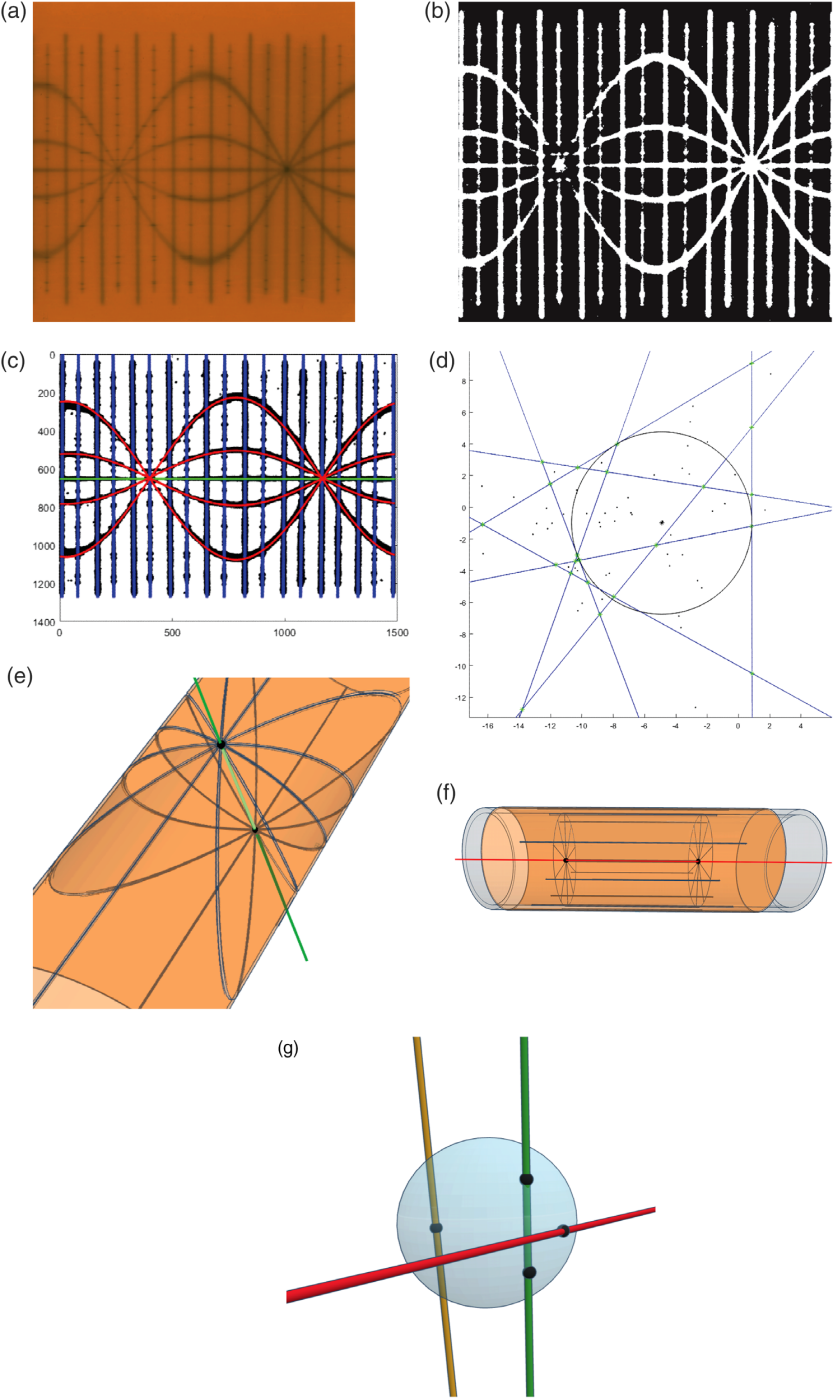
(a) The slit exposures on film approximate cylindrical sections. When the film is flattened, these sections will either be vertical lines, a horizontal line, or sine waves. (b) The film is binarized using threshold methods to determine if a pixel is or is not exposed. (c) The location of each exposed pixel forms the data to fit the lines and sine waves. (d) Each fitted curve is a spoke, and the smallest circle to touch all spokes is found. (e) The collimator axis has two star shots and two smallest circles. Joining the centers of the entrance circle to the exit circle makes a line that represents the axis. A similar statement applies to the couch axis. (f) The gantry forms virtual star shots on any axial slice of the cylinder. Taking a slice at each end, the smallest circle touching each virtual spoke can be found and joined center to center. This line represents the gantry axis. (g) A smallest sphere touching all three axis lines can be found. It will be tangent to at least two of the three lines

The analysis for either the real or virtual star shots follows the standard 2D star shot with the process being illustrated in Figure 2. Details are described in the Appendix. When the film is rolled flat, as in Figure 2a, the spokes for the 3D star shot are curves defined by cylinder sections. These curves will be either lines or sine waves. Figure 2b is a binarization of the film, and Figure 2c fits the lines or sine waves to the data points in Figure 2b. The fitted curves form the star shot spokes and the curves are approximately linear in the neighborhood of the axis of rotation. As in the 2D case, the smallest circle that touches each spoke can be found as an inscribed circle to one of the triangles formed by the spokes. An example is illustrated Figure 2d.

The couch and collimator have identical analysis, and for definiteness, the subsequent discussions will focus on the collimator with the understanding a similar statement holds for the couch. The collimator slits form two star shots—one where the radiation enters the cylinder and one where it exits, as illustrated in Figure 2e. Each star shot has a smallest circle touching each spoke. Connecting the centers of these entrance and exit circles defines a line in 3D that represents the collimator axis.

The gantry star shot is virtual, but the analysis is otherwise the same. Figure 2f illustrates the process for finding the gantry axis. At each end of the cylinder, take an axial slice and find the smallest circle touching all the virtual spokes. The centers of these circles can be joined with a line, and that line represents the gantry axis.

Once all three axes are defined, the smallest diameter sphere that touches each axis is found, as illustrated in Figure 2g. This sphere will be tangent to two of the axes, while the third axis cuts through it.

### 2.2 Theory

**FIGURE 3:**
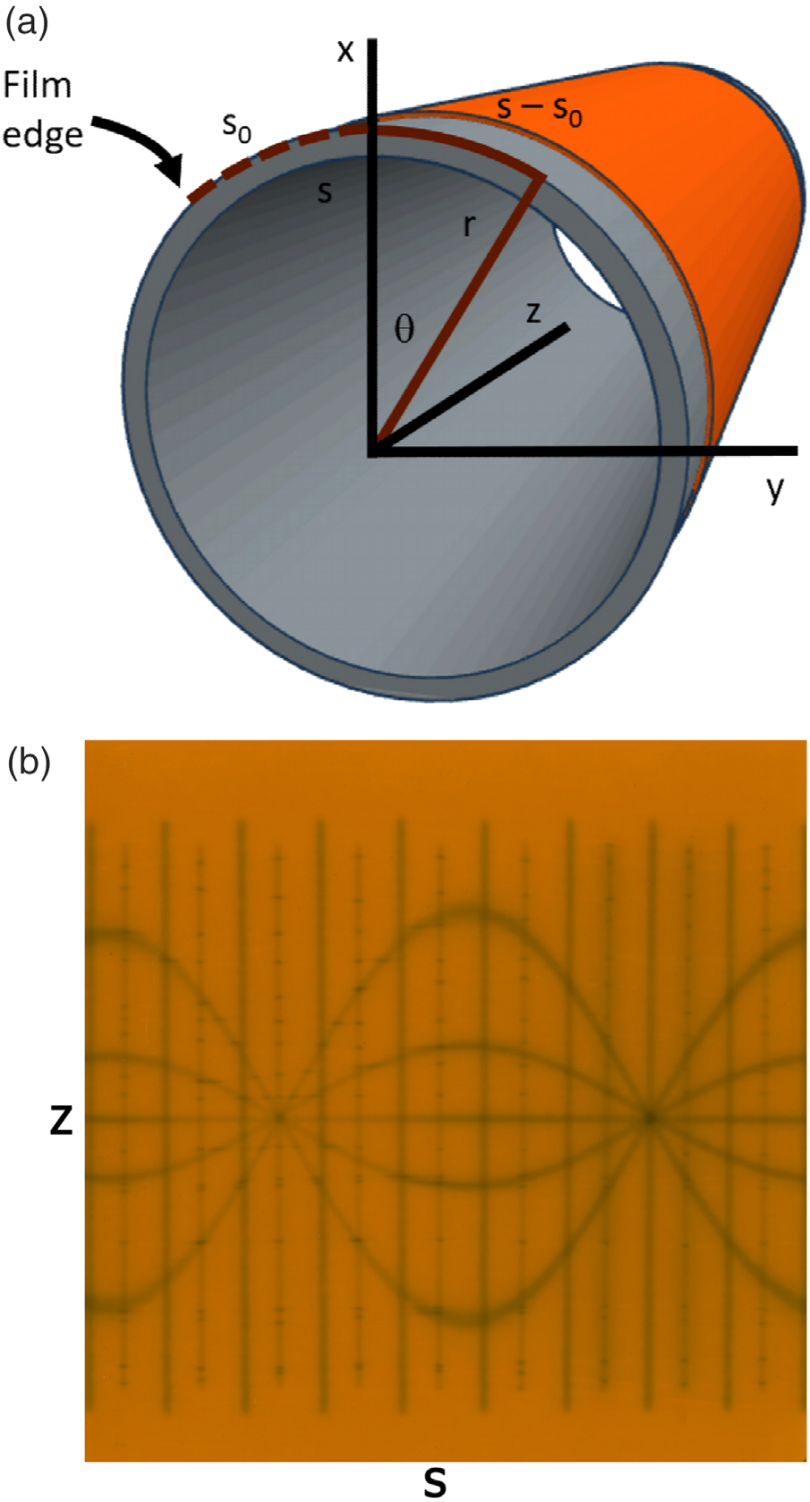
(a) Cartesian (x,y,z) and polar (r,θ,z) coordinates for the cylinder. (b) A typical film laid flat. The film has Cartesian coordinates (s,z) that relate to the arclength s around the cylinder and the position z along the cylinder's axis

The cylinder is described by both Cartesian and cylindrical coordinate systems, as shown in Figure 3. When properly aligned, points on the film relate to coordinates on the cylinder.

(1) $$z_{\mathrm{cylinder}}=z_{\mathrm{film}}$$

(2) $$s_{\mathrm{cylinder}}=s_{\mathrm{film}}$$

(3) $$x_{\mathrm{cylinder}}=r\cos\left(\frac{s-s_{0}}{r}\right)$$

(4) $$y_{\mathrm{cylinder}}=r\sin\left(\frac{s-s_{0}}{r}\right)$$

Here r is the outer radius of the cylinder, and s is the arclength measured from the edge of the film. There is an offset $s_{0}$ from the film edge to the x ‐axis such that the polar angle, *θ*, around the cylinder is given by

(5) $$\theta =\frac{s-s_{0}}{r}$$

The concept of the cylindrical section is analogous to conic sections. Apostol and Mnatsakanian^13^ give a thorough discussion of the mathematics of cylindrical sections. Each section defines either an ellipse, a circle, or line pairs on the cylinder's surface. Unwrapping the film and laying it flat, an elliptical section becomes a sine wave, the circular section becomes horizontal line, and the paired lines become a pair of vertical lines. The sine waves are related to the arclength, the radius, and the angle of inclination β that the cutting plane takes against the cylinder by

(6) $$z\left(s\right)=h\sin\frac{s-s_{0}}{r}$$

(7) h=rtanβ

The angle of inclination is the complimentary angle to the collimator or couch angle.

### 2.3 Procedure

**FIGURE 4:**
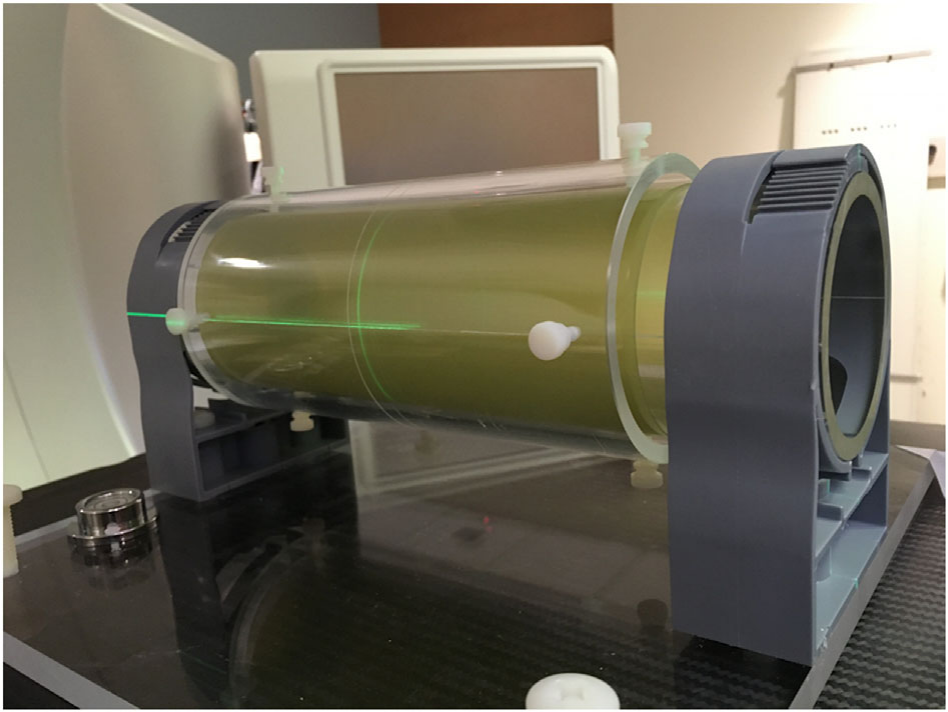
3D Star shot phantom

A cylindrical phantom, seen in Figure 4, was designed using Rhinoceros 7 (Robert McNeel and Associates) and was built to accommodate a single sheet of Gafchromic EBT3 8″ × 10″ film. The phantom has an inner and an outer cylinder. The film mounts to the outside surface of the inner cylinder, and this cylinder is inserted into the outer cylinder. The outer cylinder acts as buildup for the film, an anchor to steady the film, and a mechanical system to align the phantom with the linac's mechanical/optical axes by using markings etched into the outer cylinder's surface.

Exposures were taken on a TrueBeam accelerator (Varian Medical Systems, Palo Alto, CA). The axes of the cylinder were aligned to the mechanical axes of the linac. The multileaf collimator (MLC) was narrowed to a 1‐mm slit width and 300 Monitor Units (MU) delivered per slit. The outer cylinder acted as build up material and dramatically reduced the MU required to blacken the film. Nice gantry exposures can be taken by picking the incrementing gantry angle as $\Delta G\;=360^{\circ }/\left(2n+1\right)\;$. These angles choices (120°, 72°, 40°, or 24°) have the property that the entrance and exit exposures are evenly spaced at ΔG/2. The 40° was used in this protocol, producing a line on the film every 20°. The collimator and couch slit exposures are readily distinguishable if the collimator exposures are taken with a nonvertical gantry angle. However, these were taken with gantry angle 0°, and consequently the couch and collimator exposures became confounded with each other. Alternating spokes between couch and collimator facilitated tracking which spoke was which. The protocol used collimator angles at 0°, 30°, 60°, 90°, 330°, and 300° and the couch angles at 15°, 45°, 75°, 285°, 315°, and 345°.

The process to analyze the film is illustrated in Figure 2. Fewer couch and collimator angles were used in this film to facilitate visualization for the explanatory figures. In‐house software was written in MATLAB (The Math Works, Inc. MATLAB. Version 2021a) to analyze a film. The analysis is described in the Appendix.

## 3 RESULTS

**FIGURE 5:**
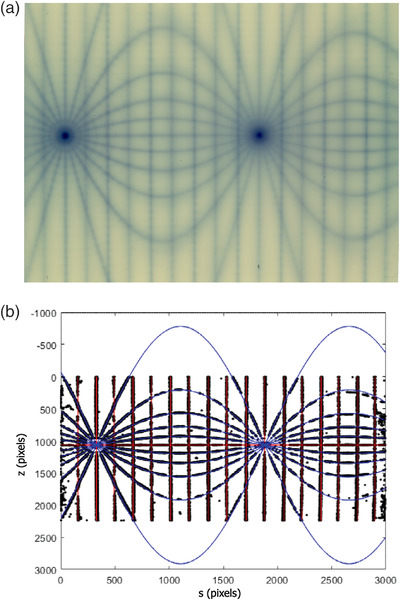
A 3D star shot following protocol. (a) Film was exposed and scanned. (b) Curves were fitted to the binarized data. Notice some of curves are drawn off the edge of the film

The methods described above were applied to a clinical protocol using 6 collimator angles, 6 couch angles, and 9 gantry angles. The results are shown in the Figure 5 and Table 1.

**TABLE 1:** Results for the film in Figure 5

Smallest sphere	
Diameter	0.5085 mm
Center P	(−0.25, 0.042, 0.19) mm
Distance from P to gantry axis	0.2543 mm
Distance from P to couch axis	0.2543 mm
Distance from P to collimator axis	0.2353 mm

The center of the sphere is point P located, in mm, at (−0.25, 0.042, 0.19). This point represents the nominal isocenter of the machine relative to the center of the cylinder phantom. If the cylinder were perfectly aligned to the isocenter, then the sphere would be at (0, 0, 0). Since the cylinder is set up at each experiment, this result will vary from measurement to measurement. Small deviations are acceptable, but there is no specification for how close this value needs to be. Typically, one would expect less than a 1 mm. The diameter of the sphere was 0.51 mm, and CPQR's annual test A15^7^ specifies this should be less than 1 mm.

## 4 DISCUSSION

There are multiple advantages to this methodology. First, once the in‐house software was completed, the time to setup, execute, and analyze a 3D star shot was comparable to that for a 2D star shot. Second, using a single film is cost‐effective, and this film can be digitized with a standard film scanner. This is equipment most cancer centers readily have. Third, the cylindrical phantom was easily designed and built in the machine shop with raw materials costing under $200. Lastly, the 3D star shot technique satisfies the requirements for CPQR's protocol and is a valuable tool to confirm the isocenter for stereotactic treatments. Moreover, this method provides a foundation for future QA requirements. For example, this technique can find the collimator axis of rotation for any fixed gantry angle, and the motion of the isocenter could be tracked in three dimensions as the gantry angle rotates.

**FIGURE 6:**
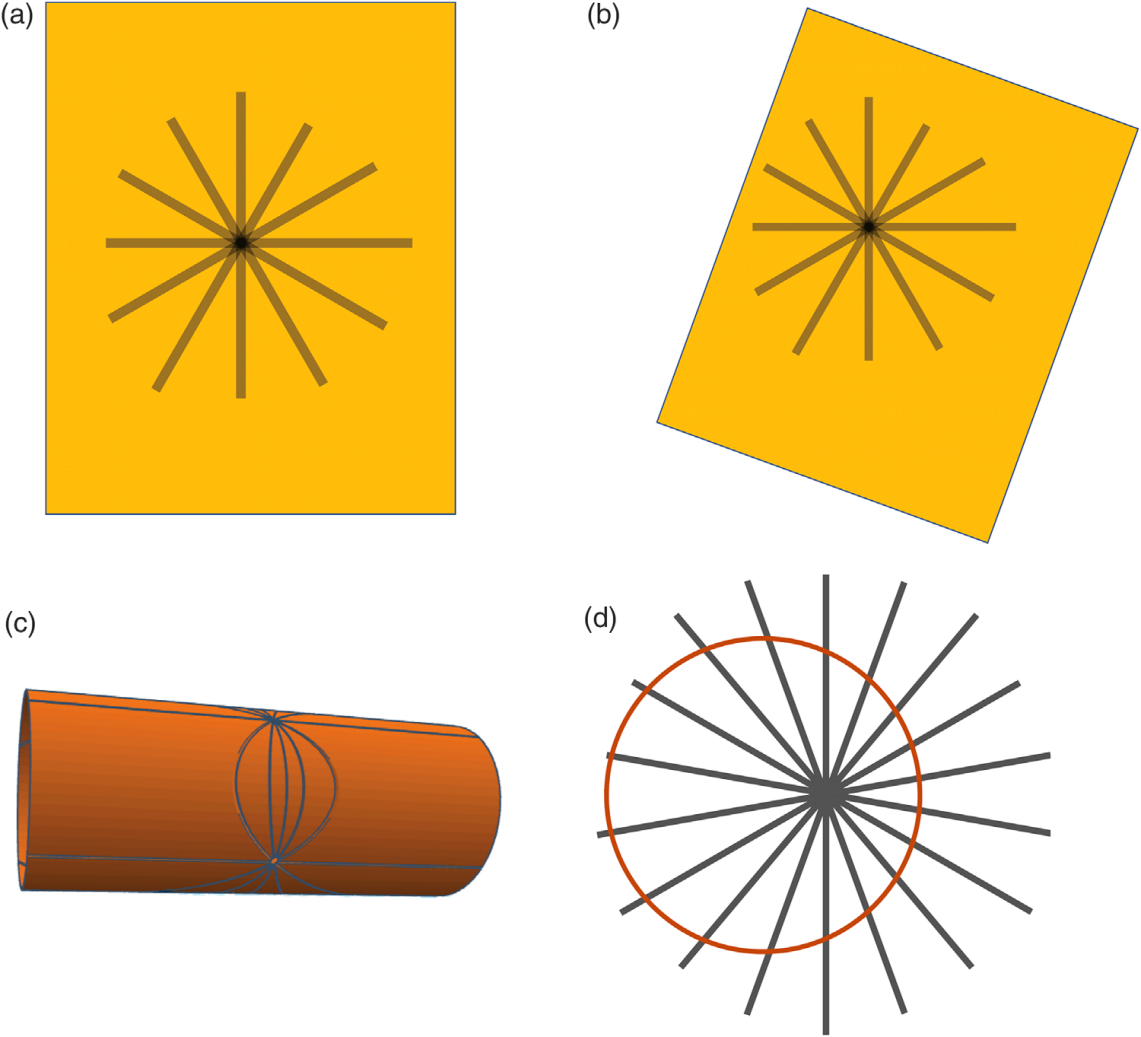
(a) 2D star shot where the film is square to the ordinate axes of the linac. (b) 2D star shot where the film was not carefully aligned but the exposed star pattern is identical to that in the carefully aligned film in (a). Both films give the same diameter circle. (c) A 3D collimator star shot where the center of the cylinder is not at the isocenter. The entrance and exit star patterns have moved closer to each other. (d) The gantry slit exposures will have unequal spacing between the lines

All current limitations of the 3D star shot could be resolved with improved, robust software. First note that the results are independent of the film alignment. This is analogous to the 2D star shot, as illustrated in Figures 6a and 6b. Here, the star pattern does not change when the film is shifted and rotated. The smallest circle touching each spoke has the same diameter regardless of the film orientation. The 3D case is also not sensitive to the orientation of the film/cylinder. Figure 6c and 6d shows what happens when the center of the cylinder is shifted relative to the isocenter. The cylinder sections are still ellipses, but the entrance and exit stars are no longer on opposite sides of the cylinder. Likewise, the gantry exposures will still be lines but these will not be evenly spaced around the cylinder.

Additional changes occur if the cylinder z‐axis is rotated relative to the gantry axis. In this circumstance, a gantry exposure becomes a sine wave, belonging to a very elongated elliptical cylinder section. The horizontal line will also become a small‐amplitude sine wave.

**FIGURE 7:**
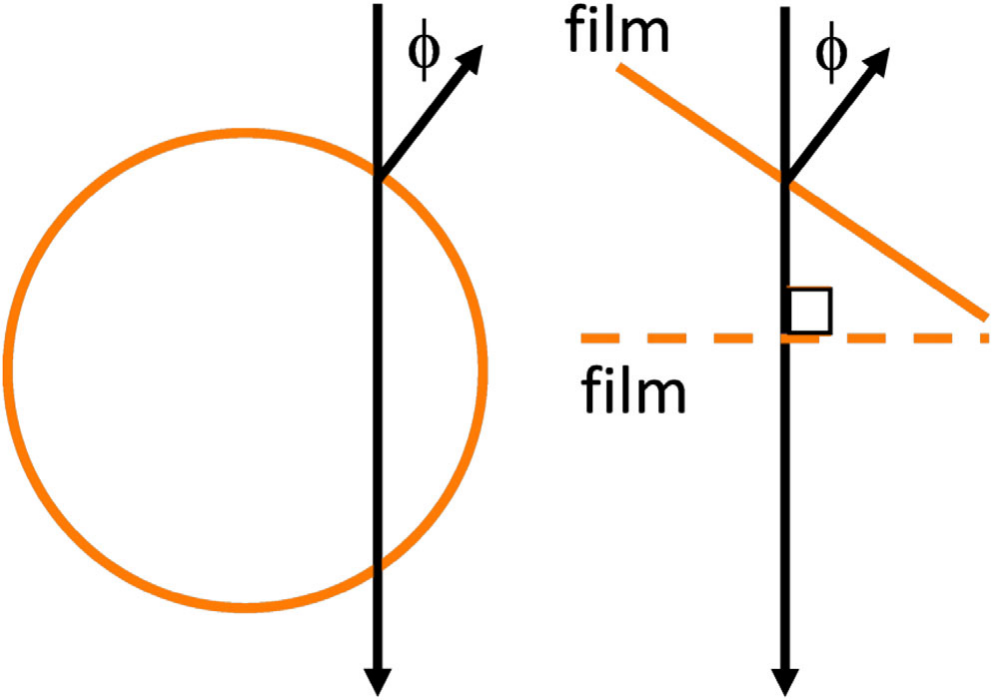
When the film normal makes an angle ϕ to the axis of rotation, the film needs to be projected to a plane perpendicular to the axis of rotation and the smallest circle found on this plane

If the normal to the film is not parallel to the axis of rotation, the star becomes distorted. For this situation, illustrated in Figure 7, the goal is to either find a smallest ellipse instead of a circle or to map the film points on to a plane perpendicular to the axis of rotation and find a smallest circle. The ellipse on the film relates to the circle on the virtual film plane. One axis of the ellipse will equal the radius of the circle, while the other is the radius divided by cos ϕ.

All of these alignment effects can be accounted for most readily in 3D. Each exposed pixel can be mapped to a point on the surface of the cylinder, and the 3D point cloud then fitted to planes. The smallest circle touching each spoke can be changed to finding the smallest sphere touching each plane.

A phantom with nearly identical functional design was independently developed by Song.^14^ They too use a two‐layered cylinder, but they do not use a star shot technique. They employ small fields instead of slits, and a small sphere located at the center of the cylinder. Their test is like the Winston‐Lutz test, and they use couch and gantry combinations to achieve noncoplanar beams.

## 5 CONCLUSIONS

A technique to measure the location of each axis of rotation relative to one another in 3D was presented. It is an extension of the 2D star shot except the 3D star shot wraps a film on a cylinder surface, and slit exposes of the film are taken for all three axes of rotation. It has the advantage of using a single sheet of film and film scanner. The 3D star shot measurement satisfies the requirement for CPQR guidelines and provides confidence in the linac's performance to support advanced treatment techniques such as stereotactic radiosurgery.

## 6 APPENDIX

The segmentation of analysis of a 3D star shot film can be broken up into the following tasks:

1. Binarization
2. Curve fitting
3. Circle inscription
4. Axis line representations
5. Sphere inscription

### 6.1 Binarization

Gray scale information is not required other than to decide which pixel was exposed, and which was not. The coordinates of the exposed pixels are used for data in the curve fitting routines. Dynamic thresholding^15^ was implemented to binarize the image into exposed and unexposed pixels. This technique creates a blurred background image using a Gaussian filter with a standard deviation equal to the slit width. Let (z,s) denote the row and column coordinates of a pixel, and let $g_{z,s}$ be grayscale value of the image, and $b_{z,s}$ be the grayscale value of the blurred background image. A threshold δ>0 was picked, and for dark objects in the image, the binarized image set is

(8) $$B=\left\{\left(z,s\right)|\left(g_{z,s}-b_{z,s}\right)\leq -\delta \right\}$$

### 6.2 Curve fitting

This needs to address two problems. First the data needed to be categorized as to belonging to one curve or another. There were vertical lines, a horizontal line and sine waves all on the same image and exposed pixels had to be assigned to each of these. Once data are categorized, the curves were fitted one curve at a time. The Matlab function polyfit worked well for the lines, and a small function to fit the sine waves was written using the fit function.

**FIGURE 8:**
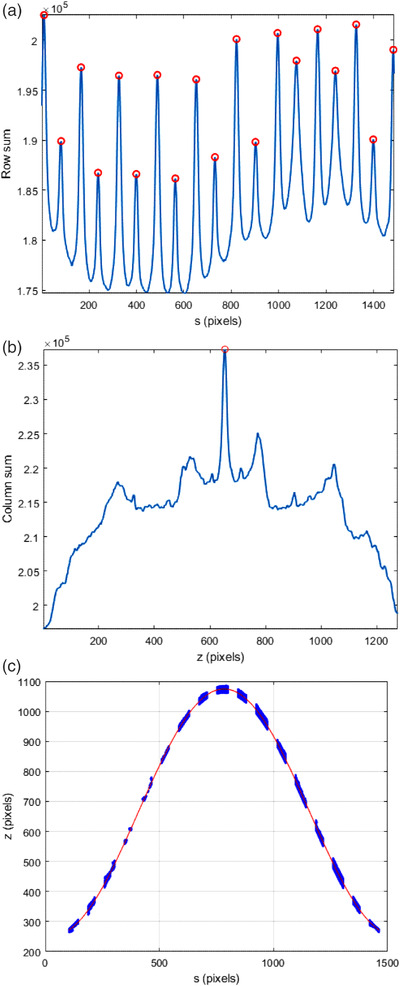
(a) Summing each column identifies which columns are in the proximity of a vertical line. A strong peak appears near a vertical line because every point in the column is scored as an exposed pixel. (b) Summing each row identifies which row is in the proximity of the horizontal line. A strong peak occurs near the horizontal line because every point in the row is scored as an exposed pixel. (c) When pixels associated with the vertical lines and horizontal lines are removed from consideration, the remaining exposed pixels are associated with families of sinewaves. The associated exposed pixels to a single sinewave are selected by specifying the angle of inclination β. Once the pixels for a given curve are selected, they can be fitted to a sine wave

The main challenge was categorizing the data. Vertical lines were categorized by summing the rows for each column. A plot the column sum versus column number is shown in Figure 8a, and identifiable peaks occur near each vertical line. Let p be the column number of a peak. The points associated with this line were found by searching a narrow strip about p.

(9) $$V_{p}=\left\{\left(z,s\right)|\;\left|s-p\right|\leq \varepsilon \right\}$$

A similar technique was done for the horizontal line. The row sum versus row number is shown in Figure 8b. If the highest peak is located at row t then the points associated with the horizontal line are found by searching a narrow strip about t:

(10) $$H_{t}=\left\{\left(z,s\right)|\;\left|z-t\right|\;\leq \sigma \right\}$$

As defined here, $H_{t}$ has points in common with the different $V_{p}$ ’s. This overlap was subtracted out of $H_{t}$ to avoid double counting.

Sine wave data points were categorized by taking advantage of known information. The angle of inclination β is the complimentary angle to the collimator or couch angle and is a known value by the observer who exposed the films. Equations (6) and (7) predict values $\hat{z}$ for each s, and a narrow strip, characterized by ξ, can be drawn about $\left(\hat{z},s\right)$. This strip classified points near the sine wave associated with the inclination β:

(11) $$S_{\beta }=\left\{\left(z,s\right)|\;\left|z-\hat{z}\right|\;\leq \xi \right\}$$

Points in $S_{\beta }\cap V_{p}$ and $S_{\beta }\cap H_{t}$ were removed from $S_{\beta }$. A typical example is shown in Figure 8c.

One last detail focused on points assigned to one curve that might be better assigned to another. The lines and sine waves found from the above process were used to reclassify each point according to which curve was closest to it. Once all points were reclassified, the final curves were fitted to the newly assigned data.

### 6.3 Inscribing a circle

The local behavior of each fitted curve near the intersections determines what is the smallest circle to touch each spoke. Each sine wave was approximated by line using its first order Taylor series expansion in this neighborhood. The problem now reduced to the same problem for the 2D star shot of finding the smallest circle to touch all lines. Take any three lines. These form a triangle that has a unique inscribed circle. Geometry determines the center and radius of the circle. The largest of these inscribed circles was the smallest circle that touched every line. For example, incrementing the gantry in 40° steps gives 9 lines and 84 triangles to examine. A typical result is plotted in Figure 2d, and close inspection shows points plotted between the lines. These points are the centers of the 84 circles. Only the largest circle was plotted. This circle was tangent to three lines, with all other lines crossing the interior of the circle.

### 6.4 Axis representation

The couch exposures produced two star shot images on opposite sides of the cylinder. One star shot was an entrance exposure, and the other was an exit exposure. Each star shot has a circle, and joining their centers creates a line representing the couch axis. Similar statements apply to the collimator exposures. The gantry's virtual star forms on any axial slice. Axial slices were taken at each end of the cylinder to give the two star shots and subsequently two circles. The line connecting their centers represents the gantry axis.

### 6.5 Inscribing a sphere

Finding the smallest sphere to touch three noncoplanar lines was framed as an optimization problem to be solved with Matlab. The solution sphere is typically tangent to two of the three lines, while the third line cuts through the sphere, as shown in Figure 2g. There is a possibility that the sphere may be tangent to all three lines. To find the sphere's center, pick an arbitrary point P in 3D space. Compute the distance from P to each axis line and take the maximum of these three distances. This maximum was the score value that Matlab's optimization routine minimized by moving P.

## AUTHOR CONTRIBUTIONS

Robert Corns developed concepts, involved in data collection and analysis, and was principal author. Kaida Yang designed and fabricated phantom. Shiva Bhandari and Makunda Aryal were in involved in analysis process. Kaida Yang, Mason Ross, Peter Ciaccio, Shiva Bhandari, and Makunda Aryal involved in data collection and contributed to writing/editing article.

## CONFLICT OF INTEREST

The authors declare that there is no conflict of interest that could be perceived as prejudicing the impartiality of the research reported.
